# Efficacy and Safety of Prostatic Artery Embolization in the Treatment of High Risk Benign Prostatic Hyperplasia and its Influence on Postoperative Life Quality of Patients

**DOI:** 10.3389/fsurg.2022.905394

**Published:** 2022-05-17

**Authors:** Kun Wang, Ming Chen, Yiqing Liu, Weiren Xiao, Yonghong Qian, Xu Liu

**Affiliations:** ^1^ Department of Urology, The First Chinese Traditional Hospital of Changde, Changde City, China; ^2^ Vascular intervention department, The First Chinese Traditional Hospital of Changde, Changde City, China; ^3^ Department of Internal Medicine, Changde Geriatric Hospital, Changde City, China

**Keywords:** benign prostatic hyperplasia, prostatic artery embolization, high risk, prostatic artery, quality of life

## Abstract

**Objective:**

To evaluate the efficacy, safety and postoperative quality of life of high risk benign prostatic hyperplasia (BPH) patients treated with prostatic artery embolization.

**Methods:**

34 patients with high-risk BPH were selectedfrom January 2020 to June 2021 in our hospital. All patients were treated with prostatic artery embolization. The changes of international prostate symptom score (IPSS), prostate volume (PV), remaining urine (RU), maximum urine flow rate (Qmax), quality of life scale -74(GQOLI-74), time to sleep without disturbance (HUS) judgment, self-rating anxiety scale (SAS) score and self-rating depression scale (SDS) were compared before operation, 1 month and 6 months after operation.

**Results:**

Prostatic artery embolization was successful in all 34 patients, including unilateral embolization in 15 patients and bilateral embolization in 19 patients. No severe complications occurred in the postoperative patients. The IPSS, PV and RU levels of the patient one month and six months after surgery were lower than those before surgery, while the Qmax level was higher than that before surgery. Besides, the IPSS, PV and RU levels six months after surgery were significantly lower than those one month after surgery, and the Qmax level was significantly higher than that one month after surgery (*p *< 0.05). The GQOLI-74 score six months after surgery was significantly higher than that before surgery (*p *< 0.05). The HUS of the patient six months after surgery was significantly increased, and the SAS and SDS scores were significantly decreased as compared with those before surgery (*p *< 0.05).

**Conclusion:**

For high-risk patients with BPH, prostate embolization is an effective and safe method, which can significantly improve the quality of life of patients after surgery and has good application prospects.

## Introduction

With the accelerated aging of the population, the incidence of benign prostatic hyperplasia (BPH) is increasing among elderly men in China (1). Histologically, BPH is mainly characterized by hyperplasia of prostatic interstitial and glandular components, anatomically, enlarged prostate, which often leads to lower urinary tract symptoms, such as dysuria, frequent urination, urinary incontinence, etc (2, 3). Long-term development of the disease will cause serious damage to the bladder and kidney function, and have many adverse effects on patients’ quality of life. In clinic, internal medicine treatment effect of some patients with benign prostatic hyperplasia is unsatisfactory, and they can not receive surgery (4, 5). In recent years, the development of surgical equipment, such as plasma and laser, has greatly improved the safety of prostate surgery, but there are still clinical complications. Especially some elderly and high-risk patients with high surgical risk can only accept indwelling catheter or cyctostomy for a long time. On the one hand, the quality of life can not be guaranteed, and on the other hand, the risk of infection is increased (6–8).

Therefore, finding a surgical approach with higher safety and ideal therapeutic effect is still the research focus in the treatment of BPH. Prostate artery embolization is a kind of interventional therapy that has appeared in recent years. The procedure includes selective intubation of bilateral prostatic arteries and injection of microspheres into both sides to realize prostatic artery embolization. It has the advantages of less bleeding, low incidence of complications during and after operative and outstanding therapeutic effect (9, 10). In this study, we treated high-risk BPH patients with prostatic artery embolization, aiming to explore the efficacy of this surgical method in the treatment of patients and the impact of related laboratory indicators, so as to provide a theoretical basis for the selection of surgical methods for BPH.

## Data and Methods

### General Information

34 patients diagnosed as high-risk BPH in our hospital from January 2020 to June 2021, aged 74–85 years old, with the average age of (80.46 ± 2.27) years old were selected. The patients had hypertension, old myocardial infarction, esophageal cancer, cerebral infarction, chronic obstructive pulmonary disease, coronary heart disease and other diseases, and eight cases had more than two diseases. Inclusion criteria: Patients who were at least 70 years old and diagnosed as BPH; by B-scan ultrasonography or MRI; The clinical symptoms include different degrees of dysuria, nocturia, and fine urine rheology in lower urinary tract syndrome. Combined with cardiopulmonary dysfunction, unable to tolerate routine surgery; Poor effect of medical treatment. All preoperative patients have informed consent and signed the operation consent form. Exclusion criteria: patients with prostate cancer or other malignant tumors of the urinary system; Patients with severe urethral stricture; Acute urinary infection or acute prostatitis infection; Allergic to iodine contrast agent; Severe coagulation dysfunction; Severe renal insufficiency.

### Research Methods

All patients were examined by PSA, MRI, color ultrasound of urinary system, aerodynamic test and digital rectal examination before operation. The malignant tumor of prostate, pathological urethral stricture and bladder neck outlet obstruction were excluded. Besides, IPSS, PV, Qmax, RU, GQOLI-74, HUS, SAS and SDS were performed. The preoperative database was established.

All patients were operated under local anesthesia. Routine preoperative indwelling catheter. The bladder of catheter was used to mark the position of the prostate. Take the supine position of the patient. Routine disinfection, towel laying and local anesthesia were carried out. After the right femoral artery was successfully punctured by Seldnger method, the catheter sheath was inserted into the 4f catheter. Cobra catheter which introduced 4F through the sheath was superselected to bilateral internal iliac arteries for rotational angiography to understand the source of the prostate artery. After the angle at which the prostatic artery could be clearly displayed was selected, Pro great micro-catheter was used for super-selection of the prostatic artery, and rotational angiography was performed to identify the prostatic artery, followed by embolization treatment. A suspension of polyvinyl alcohol particles (150–350 µm) was selected as the embolic agent, mixed with the contrast agent evenly and then slowly injected under digital subtraction angiography. In this process, it must be confirmed that there is no reflux to prevent ectopic embolism until blood flow to the prostate aorta stops. Internal iliac artery angiography was performed again, and after the complete embolism was confirmed, the contralateral prostate artery was concretized in the same way. After the opration, the puncture sheath was pulled out and the puncture site was locally compressed and bandaged. The local compression was carried out for 2 h, and the patient were bedridden for 6–24 h. After the operation, levofloxacin (0.5 g, 1 time /d) was orally taken for 2–3 days to prevent infection (shandong Lu Kang pharmaceutical group saite co., ltd., H20067724), and ibuprofen sustained-release capsule (0.3 mg, 2 times/d) was orally taken to relieve pain (Changchun Overseas Pharmaceutical Group Co., Ltd., H2066622) and assist with symptomatic treatments such as hydration. Patients without uroschesis before operation were all kept with urethral catheterization for 1 week, while those who had difficulty urinating and uroschesis before operation and kept urethral catheterization continuously continued to use indwelling urinary catheter for 2 weeks.

To observe the success rate of prostatic artery embolization. All patients were followed-up in the outpatient department for 6 months after operation. IPSS, PV, Qmax, RU were reexamined to assess the improvement of symptoms and curative effect of the patients, and GQOLI-74, HUS and SAS and SDS scores were applied to evaluate the quality of life of the patients and to compare the changes and complications of the patients before and after operation.

### Statistical Methods

SPSS22.0 software was used for processing. The experimental data are normally distributed, measurement data were expressed as mean standard deviation $(\bar{x}\pm s)$, and the enumeration data were expressed as (%). *t* test analysis was used for pairwise comparison of measurement data among groups, and analysis of variance was used for multi-group comparison. The count data were tested by *χ*^2^ test. The test level was *α *= 0.05, and *p *< 0.05 indicated that the difference was statistically significant.

## Results

### The patient’s Success Rate of Surgery and Perioperative Situation

Angiography of internal iliac arteries in 34 patients showed a total of 53 prostatic arteries, including 20 from the inferior vesical artery, 14 from the internal iliac artery, 13 from the internal pudendal artery, and 6 from the obturator artery. 52 prostatic arteries were successfully superselected and embolized, including 15 patients with unilateral embolism and 19 patients with bilateral embolism. One patient had a unilateral prostatic artery with severe tortuosity and no super-selective access to the microcatheter, and only unilateral prostatic artery embolization was performed. Bilateral embolization was successfully performed in the remaining 18 patients.

The urinary catheter was pulled out in 23 patients one week after operation, and all of them could urinate on their own. After retaining the urethral catheter for 2 weeks, the urinary catheter was removed from 11 patients, and 9 patients could urinate independently. However, the symptoms of the other 2 patients did not improve significantly. The indwelling urinary catheter was continued, and all patients could urinate independently after removing the urinary catheter one month after surgery.

### Patients with Postoperative Complications

Four of the 34 patients had skin color changes in the buttocks after surgery that were considered to be caused by a small amount of embolic reflux without special treatment and returned to normal 5–7 days later. 6 patients developed perineal distension pain, and the symptoms gradually relieved within 4–7 days after local hot compress physiotherapy. 2 patients had postoperative urinary tract infection with mild fever, which improved after anti-inflammatory and symptomatic treatment. 4 patients presented with low grade fever, which improved after physical cooling. No serious complications such as hematuria, bladder spasm, urinary incontinence, large-scale skin color change of pudendal and medial femoral region, and skin necrosis were found in the remaining patients.

### Comparison of Prostate Related Indicators Before and after Surgery in Patients

**Figure 1:**
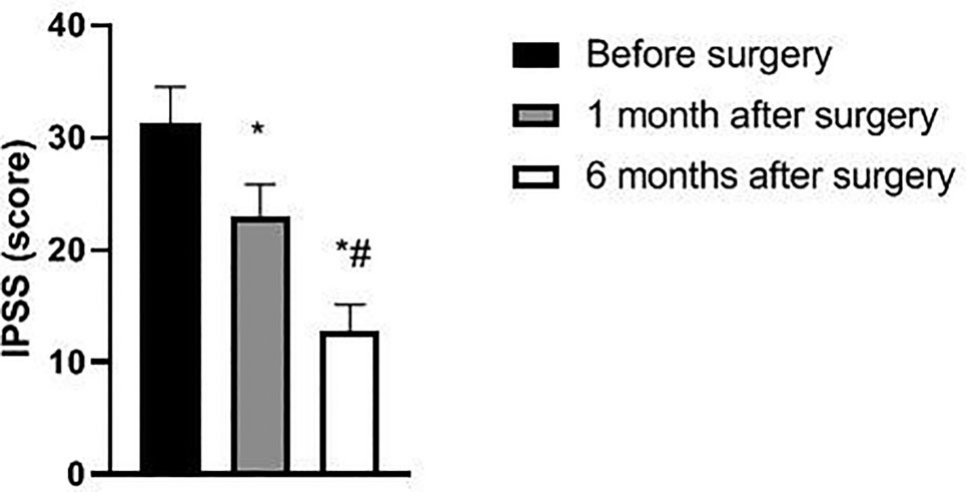
Changes in IPSS of patients before and after surgery.

**Figure 2:**
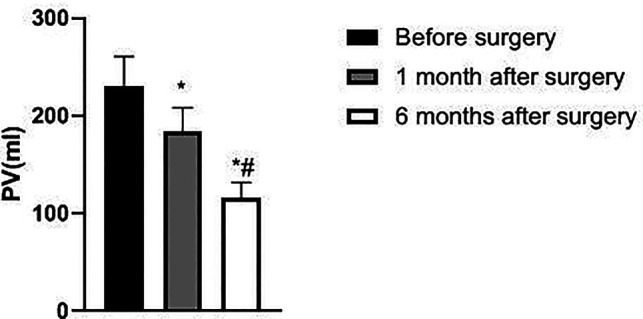
Changes in PV of patients before and after surgery.

**Figure 3:**
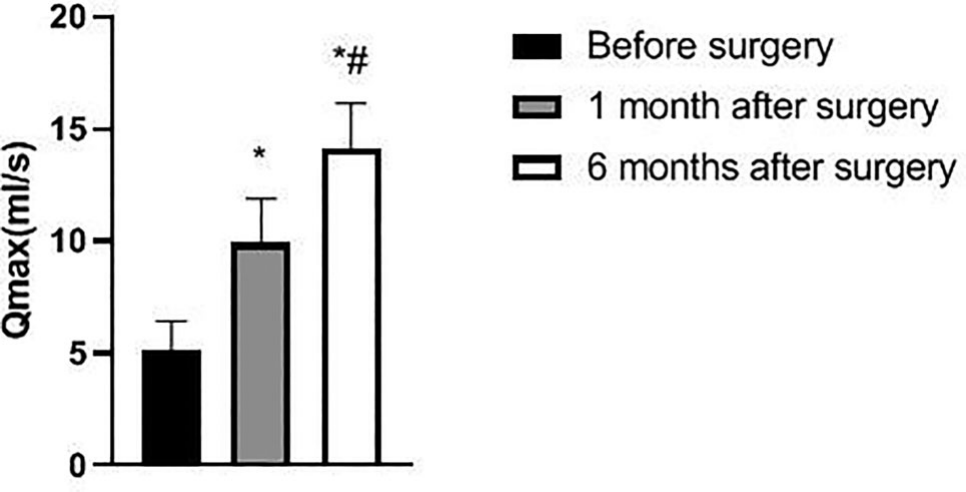
Changes in Qmax of patients before and after surgery.

**Figure 4:**
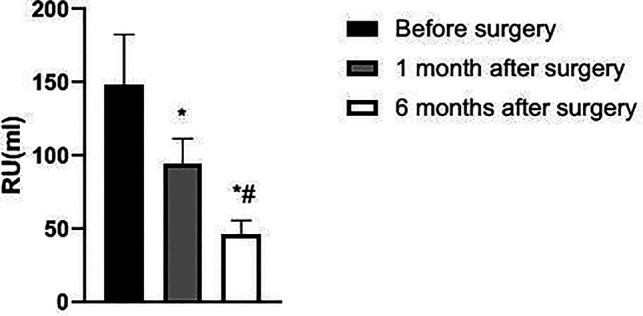
Changes in RU of patients before and after surgery.

The IPSS, PV, and RU levels of the patient at 1 and 6 months after surgery were lower than those before surgery and significantly lower at 6 months after surgery than at 1 month after surgery (*p *< 0.05). Qmax levels at 1 and 6 months after surgery were higher than before surgery and significantly higher at 6 months after surgery than at 1 month after surgery (*p *< 0.05). See Figures 1–4.

### Comparison of Patients’ Quality of Life Before and after Surgery

**Figure 5:**
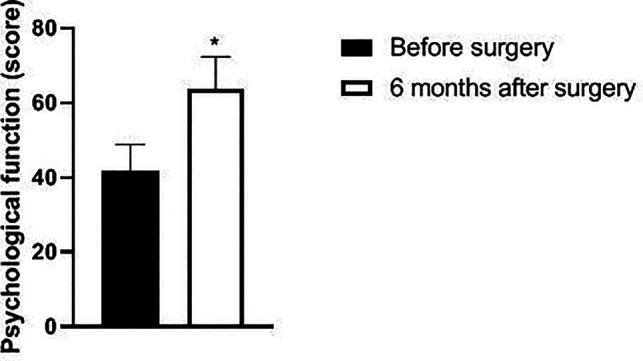
Changes in psychological function scores of patients before and after surgery.

**Figure 6:**
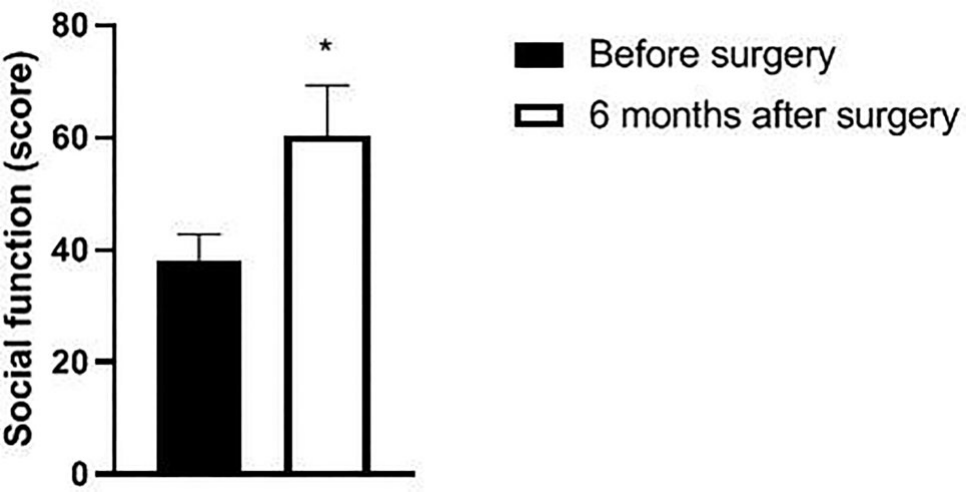
Changes in social function scores of patients before and after surgery.

**Figure 7:**
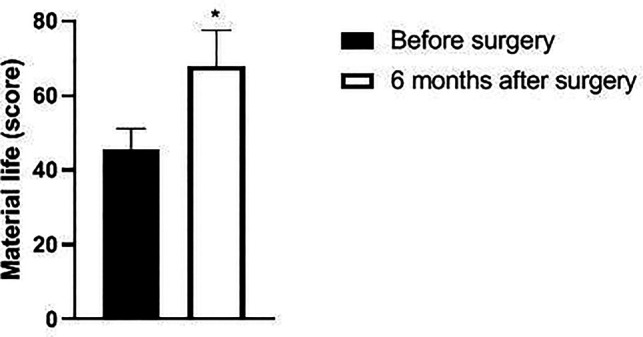
Changes in material life scores of patients before and after surgery.

**Figure 8:**
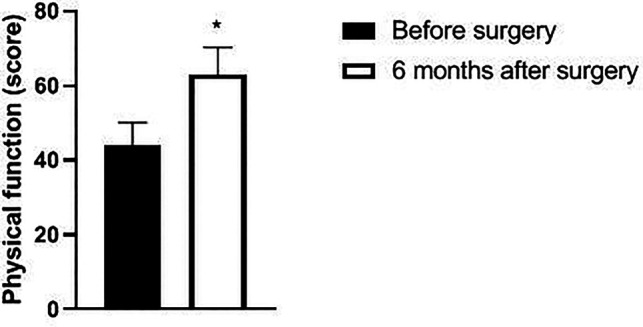
Changes in body function scores of patients before and after surgery. Note: compared with before surgery, *p < 0.05. Note: compared with before operation, *p < 0.05; compared with one month after surgery, #*p *< 0.05.

Six months after surgery, the scores of psychological function, social function, material life and physical function of GQOLI-74 were significantly higher than those before surgery (*p *< 0.05). See Figures 5–8.

### Comparison of HUS, SAS and SDS Scores of Patients Before and after Operation

**Figure 9:**
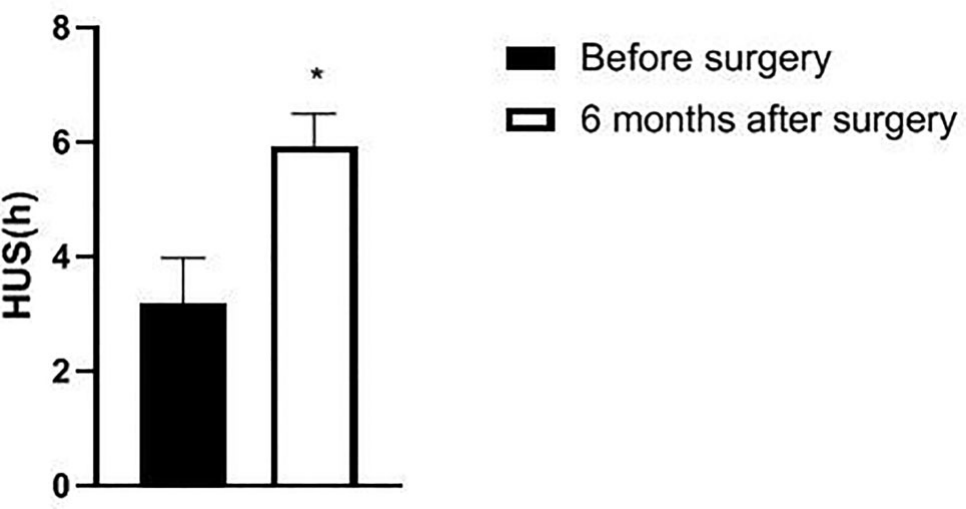
Changes of HUS in patients before and after surgery.

**Figure 10:**
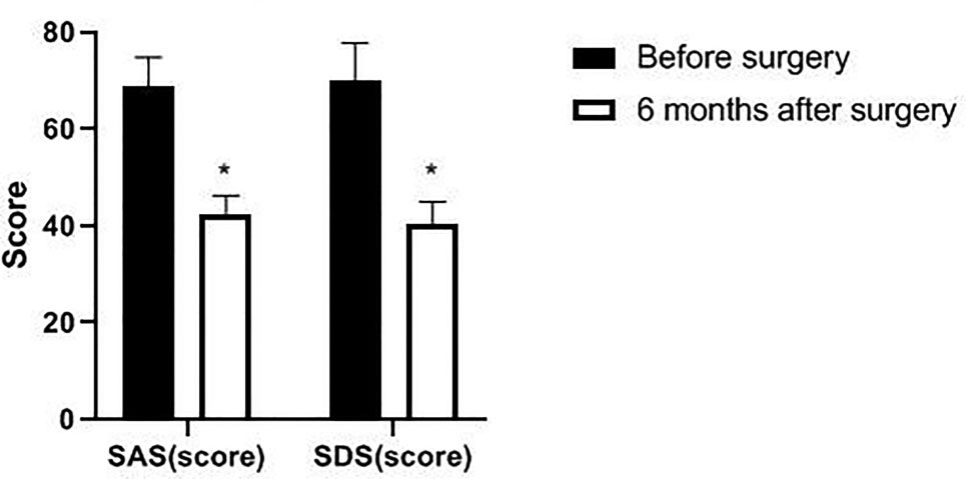
Changes of SAS and SDS scores in patients before and after surgery. Note: compared with before surgery, *p < 0.05.

The HUS of the patient six months after surgery was significantly increased, and the SAS and SDS scores were significantly decreased as compared with those before surgery (*p *< 0.05). See Figures 9, 10.

## Discussion

BPH is the most common disease in elderly men. When the disease progresses to a certain stage, patients often suffer from chronic urinary retention due to obstruction of lower urinary tract, causing lower abdominal pain, repeated urinary tract infection, hematuria, which easily lead to serious coplications such as bladder dysfunction, renal insufficiency, electrolyte disturbance, etc., and poses a serious threat to the health of elderly men (11–13). Although BPH high-risk patients have obvious symptoms of lower urinary tract obstruction and their quality of life declines, their tolerance to anesthesia and surgical intervention is poor due to physical factors. Therefore, it is the common desire of patients and doctors to find a new treatment to improve the quality of lifeof patients.

Prostate embolization, as a new treatment, can be performed under local anesthesia, with little trauma. Its principle is to use the interventional method to symbolize the prostate artery and block the blood supply to the prostate, thus leading to ischemia, hypoxia, necrosis and atrophy of some prostate tissues, alleviating the obstruction of lower urinary tract and improving the symptoms (14–16). In this study, 34 patients with high-risk BPH were operated, and all operations were successful. There were 15 patients of unilateral embolism and 19 patients of bilateral embolism, and a total of 53 prostatic arteries were embolized. Only one patient received unilateral prostatic artery embolization, because one prostatic artery was too curved and microcatheter could not selectively enter. The key of prostate embolism is the superselective prostatic artery, while high-risk patients are generally older, with arteriosclerosis and tortuosity, which increases the difficulty of catheter insertion (17). In this respect, our experience was that the use of a coaxial microcatheter during the operation to indicate the angle of good starting point of prostate artery could lead to crooked blood vessel and improved the success rate of the operation.

Prostatic arterial embolization can be completed under local anesthesia, reducing the damage to the central nervous system. In addition, it can contract the prostate by blocking the blood supply to the prostate artery, which can improve the clinical symptoms and cause less trauma to the surrounding tissues, thus contributing to postoperative rehabilitation (18–20). The results showed that all 34 patients received good curative effects, and no severe complications occurred after the operation. Some studies believe that the use of prostatic embolism in the treatment of elderly patients with BPH can alleviate the patient’s dysuria and improve the patient’s urination function. The main reason is that prostatic artery embolization under fluoroscopic guidance can improve urethral obstruction, reduce the pressure of the middle lobe of the prostate on the urethra and cause little damage to the urethral tissue, thus improving urination function and reducing the incidence of urethral infection and other complications (21–24).

This study also showed that the prostate function-related indicators of the patient at one month and six months after surgery were significantly better than those before surgery, and PV was also significantly decreased. Prostatic arterial embolization can block the blood supply of the enlarged prostate by embolizing bilateral prostatic arteries, which can induce ischemic necrosis and apoptosis of prostate tissue and block the internal circulation of androgen to the prostate tissue, thus improving the therapeutic effect through androgen-related apoptosis (25, 26). At the same time, prostatic artery embolization can effectively destroy some prostatic nerves, thus eliminating the increase in smooth muscle tension in BPH patients, thus reducing urethral resistance and increasing urinary flow (27, 28). Considering the improvement in patients’ quality of life scores and HUS, prostatic artery embolization is a recommended minimally invasive method to treat high-risk BPH patients (29).

In summary, prostate embolization is an effective and safe method with good application prospect for high-risk patients with BPH. However, due to the small sample size in this study, its long-term efficacy and safety require further observation and study.

## Data Availability

The original contributions presented in the study are included in the article/supplementary material, further inquiries can be directed to the corresponding author/s.
